# Exploring the bioaccessibility and intestinal absorption of major classes of pure phenolic compounds using *in vitro* simulated gastrointestinal digestion

**DOI:** 10.1016/j.heliyon.2024.e28894

**Published:** 2024-03-27

**Authors:** Adriana C.S. Pais, Ezequiel R. Coscueta, Maria Manuela Pintado, Armando J.D. Silvestre, Sónia A.O. Santos

**Affiliations:** a CICECO-Aveiro Institute of Materials, Chemistry Department, University of Aveiro, 3810-193 Aveiro, Portugal; b Universidade Católica Portuguesa, CBQF - Centro de Biotecnologia e Química Fina – Laboratório Associado, Escola Superior de Biotecnologia, Rua Diogo Botelho 1327, 4169-005, Porto, Portugal

**Keywords:** Phenolic compounds, Bioaccessibility, Simulated gastrointestinal digestion, Bioactive compounds

## Abstract

The bioaccessibility and bioavailability of phenolic compounds (PC) influence directly their role in disease prevention/control. Studies have evaluated this ability through complex plant and food matrices, which may reflect more a synergistic effect of the matrix than the ability of the PCs, hindering their individual exploitation in nutraceutical or pharmaceutical applications. In the present study ten pure PCs representing major classes were evaluated for their bioaccessibility and intestinal absorption in an *in vitro* simulated gastrointestinal digestion (SGD). This is the first study concerning the bioaccessibility evaluation of pure phloretin, phloroglucinol, naringin, naringenin and daidzein, while no *in vitro* SGD has been performed before for the other compounds considered here. PCs were analyzed through ultra-high-performance liquid chromatography coupled with diode-array detection and tandem mass spectrometry (UHPLC-DAD-MS^n^). Most of the compounds remained present along the gastrointestinal tract, and the bioaccessibility was in general higher than 50%, except for quercetin, epigallocatechin gallate, and ellagic acid. All compounds were highly absorbed in the intestine, with phloretin showing the lowest percentage at about 82%. The study findings provide new knowledge on the bioaccessibility and intestinal absorption of different PCs classes.

## Chemical compounds studied in this article

Quercetin (PubChem CID: 5280343), Rutin (PubChem CID: 5280805), Naringenin (PubChem CID: 932), Naringin (PubChem CID: 442428), Epigallocatechin-gallate (PubChem CID: 65064), Apigenin (PubChem CID: 5280443), Daidzein (PubChem CID: 5281708), Phloretin (PubChem CID: 4788), Phloroglucinol (PubChem CID: 359) and Ellagic acid (PubChem CID: 5281855)

## 1 Introduction

From the beginning of humankind, plants have been an essential source of food with diverse nutritional compositions and natural healthcare products [1]. The consciousness that nutrition is essential for human health and the growing knowledge about plant bioactive compounds triggered an increasing interest of the scientific community's over the last decades [2]. PCs exhibit a vast structural diversity and are widely distributed as a class of plant secondary metabolites found in human diets, including fruits, nuts, legumes, and vegetables [[3], [4], [5]]. Recently published studies have focused on these compounds due to their important biological activities, including antioxidant, anti-inflammatory, antimicrobial, and anti-proliferative activities among others [2,5]. The human health beneficials effects of quercetin (QUE) hhave been widely studied, for example to understand how its antioxidant activity can be exploited for medicinal applications [6]. Moreover, besides its known anti-inflammatory and antiviral activities [7,8], the biological activities of QUE gut microbiota metabolites have demonstrated antiproliferative activity [9]. On the other hand, rutin (RUT), a glycosylated form of QUE, showed to have anti-obesity properties through its promising prebiotic effect on gut microbiota [10]. Additionally, other phenolic compounds show also promising bioactivities, for example, apigenin (APG) (a flavone) was reported to show anti-inflammatory and anti-microbial activities [11,12] and the ability to suppress tumors through their effect on gut microbiota composition [13]; whereas ellagic acid (EA) (a phenolic acid) was shown to be a promising compound in the prevention and therapy of several diseases due to its antioxidant and anti-inflammatory activities, to its role in cancer chemoprevention, and in the prevention of cardiovascular diseases [14]. These compounds may provide preventive or therapeutic benefits for various disorders.

However, it is essential to consider the bioaccessibility and bioavailability of PCs when discussing their biological activities. The compound fraction released from the ingested food/plant matrix that becomes absorbed at the level of the gastrointestinal tract, reaching the organs and tissues through the systemic circulation, and subjected to metabolism and excretion defines bioavailability [15,16]. On the one hand, bioavailability is dependent on external factors, namely bioactive compounds' physicochemical properties (dependent on PCs structures), matrix effects (e.g., interaction with other compounds), and processing and storage conditions, among others [4,5,17]. On the other hand, three main gastrointestinal stages affect bioavailability. The first of these is bioaccessibility, defined as the amount of ingested compound released from the matrix and accessible for small intestine absorption. Thus, bioaccessibility includes liberation, solubilization, and interaction. Then, absorption (membrane transport) is the next bioavailability step, followed by transformation (chemical transformation and metabolism) [[15], [16], [17], [18], [19], [20]].

Hence, more abundant PCs were not necessarily those with higher bioavailability [17]. In general, they are less bioavailable than that micro and macronutrients [21].

PCs' beneficial health effects could be local, through the gastrointestinal tract, or systemic after absorption in the small intestine [22]. Thus, the knowledge about the amount of PCs bioavailable in the target tissue, or absorbed, could be even more relevant than their content in specific food matrices or dietary supplements [5,17]. Most researchers have focused on studying the bioaccessibility or bioavailability of PCs in complex food or plant matrices [[23], [24], [25], [26], [27]]. After digestion, those complex matrices still retain about 10% of PCs, probably due to their interaction or chemical bonding with other components [[18], [19], [20],^28^]. However, there is no relevant evidence on how these key polyphenols behave without the interaction of matrix components and differ according to the polyphenols type. Moreover, some of these studies have reported that the percentage variations throughout the gastrointestinal tract could result from higher-molecular weight compounds degradation [25,26], which hinder the understanding of the bioaccessibility of simple phenolic/individual compounds. For example, in studies using fruit extracts as matrices, ellagic acid increments in gastric and intestinal phases have been associated with the depolymerization of ellagitannins leading to the release of ellagic acid [25,26]. Furthermore, many studies have not considered the solubility of PCs as an essential factor in their bioaccessibility or bioavailability, and, in fact, the gastrointestinal tract's pH variations could influence the solubility of these compounds [16,[25], [26], [27]].

**Fig. 1:**
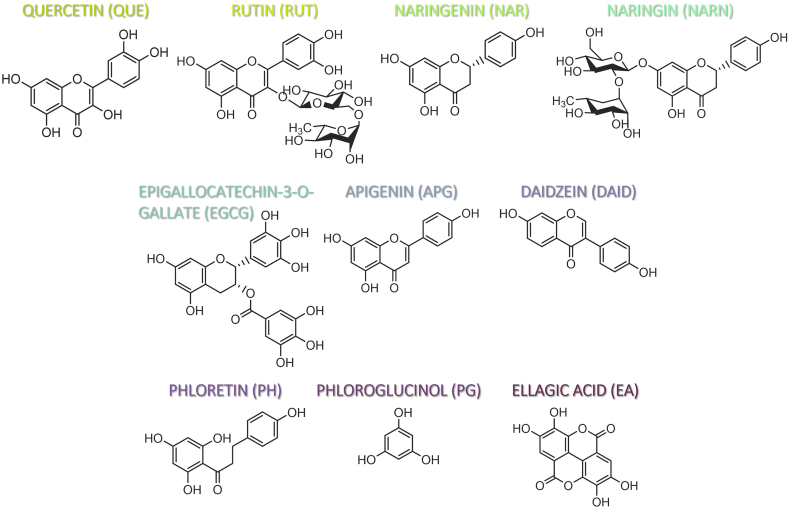
**-** Structures of selected PCs.

The present study evaluates the bioaccessibility of different PCs using INFOGEST 2.0, an *in vitro* digestion model to foods. To address the literature gap, we individually tested representative compounds from different phenolic classes (Fig. 1) to evaluate their individual bioaccessibility. These include flavonols (QUE and RUT), flavanones (naringenin (NAR) and naringin (NARN)), flavan-3-ols (epigallocatechin-gallate (EGCG)), flavones (APG), isoflavones (daidzein (DAID)), dihydrochalcones (phloretin (PH)), one monomeric tannin compound (phloroglucinol (PG)), and a phenolic acid (EA). This work represents the first study on the bioaccessibility evaluation for pure phloretin, phloroglucinol, naringin, naringenin, and daidzein. Additionally, there has been no prior *in vitro* SGD conducted for the other compounds under investigation. Moreover, we also considered the solubility of PCs, which previous studies have overlooked, as a factor that affects their bioaccessibility. Therefore, the present study may bring complementary information to that reported with food matrices, understanding the bioaccessibility of PCs in absence of other food matrices components with which they can interact. Additionally, the study of bioaccessibility of individual PCs and how they interact throughout gastrointestinal conditions may bring valuable information to be used in the preparation of formulations of supplements and/or nutraceuticals.

## 2 Material and methods

### 2.1 Materials

The phenolic standards, rutin (**≥** 95% purity, CAS No. 153-18-4), epigallocatechin-3-***O***-gallate (EGCG) (**≥** 95% purity, CAS No. 989-51-5), (+)-naringenin (**≥** 98% purity, CAS No. 67604-48-2), naringin (**≥** 95% purity, CAS No. 10236-47-2), apigenin (**≥** 94% purity, CAS No. 520-36-5), daidzein (**≥** 98% purity, CAS No. 486-66-8), phloretin (**≥** 98% purity, CAS No. 60-82-2) were purchased from Biosynth® CarboSynth (Bratislava, Slovakia); whereas quercetin (**≥** 95% purity, CAS No. 117-39-5), ellagic acid (**≥** 96% purity, CAS No. 476-66-4) and phloroglucinol (**≥**99% purity, CAS No. 108-73-6) - were purchased from Sigma-Aldrich (Madrid, Spain). HPLC-grade methanol, water, and acetonitrile were supplied by Fisher Scientific Chemicals. The enzymes α-amylase (A1031), pepsin from porcine stomach mucosa (P7012), pancreatin from porcine pancreas (P7545), and bile salts (B863) were purchased from Sigma-Aldrich (Taufkirchen, Germany). The rabbit gastric extract (RGE15) with gastric lipase activity was purchased from Lipolytech (Marseille, France). The ethanol was purchased from Merck (Algés, Portugal). A dialysis membrane with a molecular pore size of 3 kDa was purchased from Spectra/Pro (Spectrum Lab, Breda, Netherlands).

### 2.2 *In vitro* simulated gastrointestinal digestion (SGD)

Preliminary solubility tests in different solvents, namely water, sodium phosphate buffer (26.8 mM, pH 6.1), ethanol, and also a mixture of ethanol with both water and sodium phosphate buffer, were carried out for each PC (data not shown), to prevent erroneous data arising from compounds precipitation. Ethanol percentages used in these preliminary solubility tests were 10, 15, and 20% (v/v). At 10% ethanol, the solubilization of the PCs under study was not assured, whereas at 20%, the enzymatic activity was compromised. Thus, standard solutions of PCs were prepared in ethanol:water (15% v/v) at specific concentrations (Table 1), which allowed a solution without turbity or precipitates.

**Table 1:** **-** PCs solutions concentration used in *in vitro* SGD.

PC	Concentration (mg mL^−1^)
QUE	0.087
RUT	0.612
NAR	0.654
NARN	0.609
EGCG	4.13
APG	0.033
DAID	0.038
PH	0.606
PG	10.8
EA	0.047

**Fig. 2:**
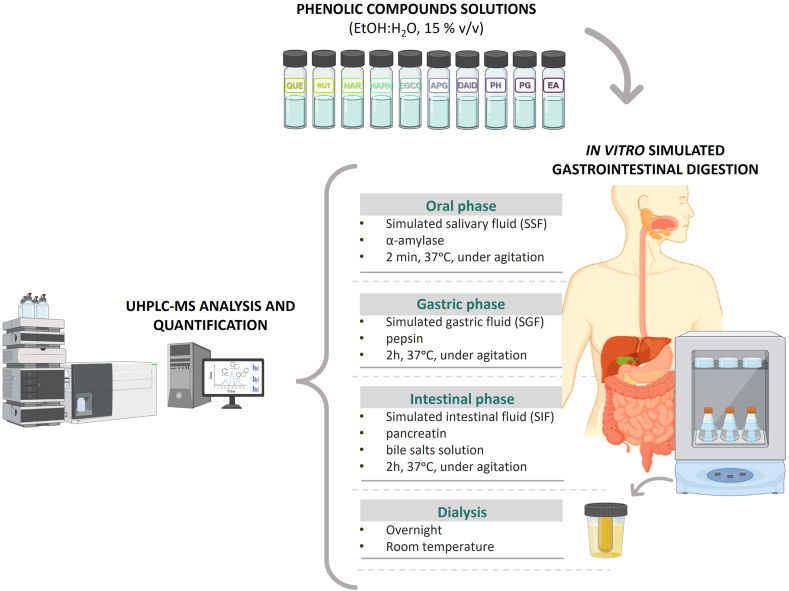
*In vitro* SGD and sample analysis and quantification by UHPLC-DAD-MSn.

Each PC solubilized in ethanol:water (15% v/v) was then submitted to an *in vitro* SGD (INFOGEST 2.0), according to a standardized protocol [29] (Fig. 2). In order to determine whether the interaction between the compounds and enzymes led to an underestimation of their content, each PC solution was submitted to the same digestion procedure, replacing the enzyme solutions with water, which were designed as controls, henceforth this will be the term used. Each sample and control were submitted to three independent *in vitro* digestion simulations, and after each digestion phase, a sample/control was collected and further analyzed and quantified by UHPLC-DAD-MSn.

Mouth digestion of each PC solution (1 mL) was diluted in 0.8 mL of simulated salivary fluid (SSF) and α-amylase (75 U mL-1). Then, the mixture was incubated for 2 min at 37 °C under agitation (200 rpm). In order to start gastric digestion, pH was adjusted to 3.0 using 1 M aqueous HCl and then, a pepsin solution (2000 U mL-1) and simulated gastric fluid (SGF) were added. Subsequently, the mixture was incubated for 2h, at 37 °C and 130 rpm orbital agitation for the stomach digestion. Finally, the small intestine digestion simulation was performed after pH adjustment to 7.0 using 1 M aqueous HCl and the addition of pancreatin (100 U mL-1), bile salts solution (10 mM) and simulated intestinal fluid (SIF), during 2 h, at 37 °C with 45 rpm orbital agitation. Natural small intestine absorption was also simulated, by placing the remaining sample in a dialysis membrane, and immersed in distilled water overnight at room temperature.

At each sampling point, an aliquot (0.5 mL) from each of the three replicates was collected. The enzymatic activity was stopped immediately by adding pure methanol to each aliquot (1:1, v/v) to promote enzyme precipitation and subsequently centrifuged at 10 000 g and 4 °C for 5 min. The supernatants were filtered with a 0.2 μm nylon filter [30]. To determine the concentration of each sample or control, it was considered the following dilution factor – 1:2, 1:4 and 1:8 for oral, gastric and intestinal digestion phase, respectively.

The bioaccessibility percentage (B%) of each PC was calculated using equation (1):

(1) $$B\left(\%\right)=\frac{\left[DS\right]}{\left[IS\right]}\times 100$$

where IS is the initial concentration of PCs in ethanol:water (15% v/v) solutions, DS is the concentration of each collected sample or control (without enzymes).

Furthermore, to determine the intestinal absorption, the aliquots collected inside the dialysis membrane were analyzed. The concentration of these samples (from inside of the membrane) corresponds to the amount of each PC that has not passed through to the outside of the membrane. Thus, to determine the percentage that has passed through the membrane, the intestinal absorption (IA%) was calculated by equation (2) both for samples and controls without enzymes as follows:

(2) $$IA\left(\%\right)=100-\left(\frac{Dialysis\;aliquot\;concentration}{Intestinal\;aliquot\;concentration}\times 100\right)$$

The values of B% and IA% were the average of the three replicates performed, with the calculated standard deviation.

### 2.3 UHPLC analysis and quantification

PCs were analyzed in a UHPLC system equipped with a variable loop Accela autosampler (set at 16 °C), an Accela 600 LC pump, and an Accela 80 Hz photo diode array detector (DAD) (Thermo Fisher Scientific, San Jose, CA, USA). Each sample was prepared in methanol:water (1:1, v/v) and filtered through a 0.2 μm nylon syringe filter. The injection volume was 20 μL, and the gradient elution method had a flow rate of 0.4 mL min^−1^, at 40 °C, using a Hypersil Gold C18 (100 mm × 2.1 mm x 1.9 μm) column protected with a pre-column (10 mm × 2.1 mm x 1.9 μm) both supplied by ThermoFisher (Thermo Fisher Scientific, San Jose, CA, USA). The mobile phase was composed of acetonitrile (A) and water:acetonitrile (99:1, v/v) (B), both with 0.1 % of formic acid. The gradient elution was performed as follows: 0–1min: 99% B; 1–17 min: 99-73% B, 17–20 min: 73-0% B, 20–25 min: 0–99% B, followed by re-equilibration of the column at 99% B for 4 min. The 200–600 nm UV spectra were recorded, and chromatograms were also obtained at different wavelengths depending on the studied compound (Table A1).

An LCQ Fleet ion trap mass spectrometer (ThermoFinnigan, San Jose, CA, USA) was coupled to the UHPLC system with an electrospray ionization (ESI) source, operating in negative mode, as previously described by Santos et al. [31].

For each PC, a UHPLC-UV standard curve was obtained. The solutions were prepared in HPLC grade methanol:water (1:1 v/v) with at least five concentrations. Each PC was quantified using the corresponding linear regression equation (Table A1). Each sample was injected in duplicate.

### 2.4 Statistical analysis

The statistical analysis for the study was carried out using OriginPRO v9.6.5.169. We first checked whether the data followed a normal distribution. In cases where we confirmed a normal distribution, we assessed the homogeneity of variances using three tests: Barlett, Cochran's, and Levene's tests. A sample was considered homogeneous variance if at least two of the three tests indicated it (*p*-value >0.05).

For samples with normal distribution and homogeneous variance, we compared the calculated B% for each SGD phase of each PC through a one-way ANOVA. To determine multiple separations of the means, we applied the Bonferroni posthoc test. However, we opted for the non-parametric Kruskal-Wallis test for cases where the data did not follow a normal distribution.

To compare the B% and IA% of samples and controls from the same gastrointestinal digestion phase, a two-sample *t*-test at the 5% probability level was performed. Three replicates of each sample/control were analyzed, except for QUE and DAID samples and EGCG, NAR (intestinal phase) and DAID controls, due to losses during the INFOGEST process. The probability level was set at 5% for every test.

## 3 Results and discussion

As mentioned earlier, due to their lower water solubility, PCs were solubilized in a mixture of ethanol:water (15% v/v). Through preliminary solubility tests, we selected this percentage as a compromise to solubilize the PCs selected (at the concentrations shown in Table 1) without affecting enzymatic activity.

### *3.1* *In vitro* simulated gastrointestinal digestion (SGD)

**Fig. 3:**
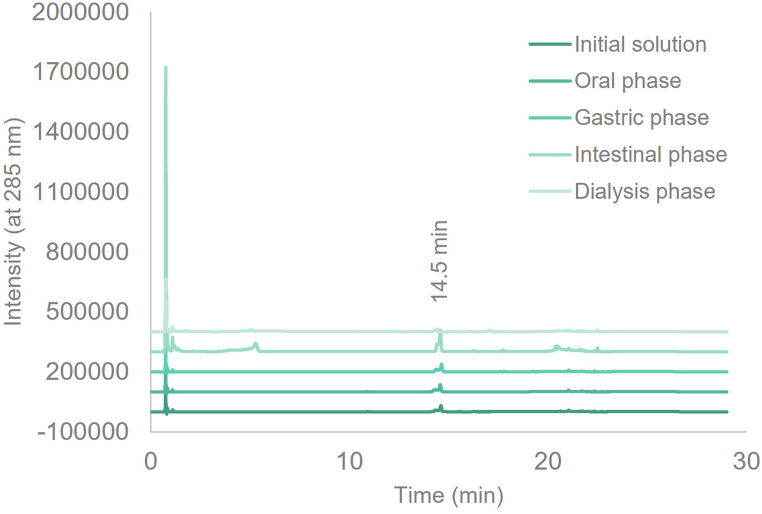
UHPLC-DAD-MS^n^ chromatograms (acquired at 285 nm) of naringin (NARN) initial ethanol:water (15% v/v) solution and collected samples after the simulated oral, gastric, and intestinal digestion and dialysis (Rt = 14.5 min).

PCs solutions underwent *in vitro* SGD and a control treatment (without enzymes). At each stage, samples were collected and analyzed using UHPLC-DAD-MS^n^ to determine the concentration of the target compound, bioaccessibility, and intestinal absorption and infer the formation of PCs metabolites. In the case of EA samples and controls the concentrations were below the limits of detection (LOD) or quantification (LOQ) during almost the entire SGD and dialysis samples. The same was observed for controls of QUE, EGCG, and DAID, as well as the APG dialysis sample. The chromatographic profile of each studied PC did not change along the SGD phases (oral, gastric, intestinal, and from dialysis) when compared with the initial ethanol:water (15% v/v) solution, as illustrated in Fig. 3 for NARN (acquired at 285 nm). Moreover, the digestion did not yield any metabolites of the studied PCs except for RUT, which produced a metabolite after intestinal digestion simulation. In this case, we detected a chromatographic peak with a molecular ion corresponding to the loss of a rhamnose unit ([M-rha-H]^-^ = 463), although the peak area of this metabolite was below the LOQ.

This suggests that the digestive enzymes did not degrade these compounds or that the resulting metabolites were in low concentrations. It is also possible that any potential metabolites had a molecular weight too low to be detected by MS or were undetectable by DAD. Overall, the above results indicate a lack of significant degradation of PCs during *in vitro* SGD.

**Fig. 4:**
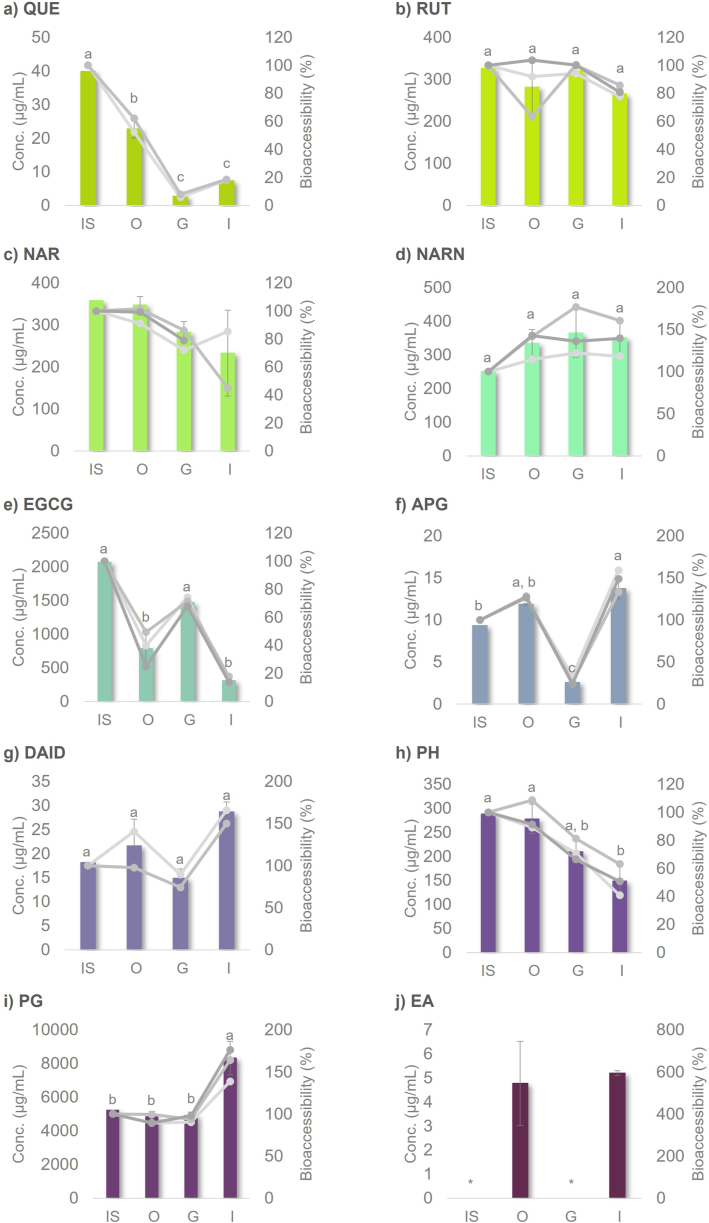
Concentration (μg mL^−1^, represented as bars) and bioaccessibilities (B%, represented each replicate as a line) of a) quercetin (QUE), b) rutin (RUT), c) naringenin (NAR), d) naringin (NARN), EGCG, f) apigenin (APG), g) daidzein (DAID), h) phloretin (PH), i) phloroglucinol (PG) and j) ellagic acid (EA) through the *in vitro***SGD**. Different letters indicate significant differences (*p* < 0.05), determined by one-way ANOVA followed by Bonferroni's post-hoc test. * < LOD and/or LOQ. IS – initial solution, O – oral phase, G – gastric phase, I – intestinal phase.

**Fig. 5:**
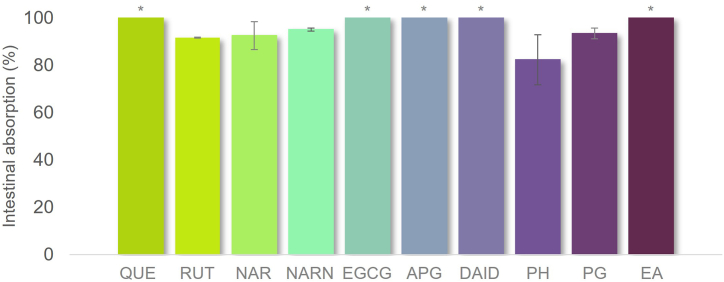
IA% of the different PCs after *in vitro* SGD. Those with an "*" were considered as 100% since the samples collected from inside the dialysis membrane were < LOD and/or LOQ, so not possible to determinate.

As for B%, shown in Table A.2 and Fig. 4, Fig. 5, the studied PCs, belonging to several PCs' classes, showed distinct behaviors along the SGD. Concentration and B% of flavonol and flavanone aglycones (QUE and NAR, respectively) after simulated digestion were lower than those of their respective diglycosides (RUT and NARN, respectively). In fact there was a substantial decrease in both concentrations and bioaccessibility of NAR and especially of QUE through the *in vitro* digestion. This could be due to the lower solubility of the aglycone forms in aqueous media, compared to glycosylated counterparts [32]. Furthermore, previous studies have reported enzymatic hydrolysis of sugar moieties from RUT and NARN by either digestive or microbial enzymes [33]. However, we have not observed the enzymatic hydrolysis of those two compounds in the present study, and their B% were not significantly different along the gastrointestinal tract.

EGCG, an ester-type compound belonging to the flavan-3-ols class, showed a significant variation along the SGD, although no metabolites were detected, which means that it was not transformed but was lost due to the changes throughout the gastrointestinal conditions (pH, enzymes and bile salts), for example, due to a variation of solubility with the pH of each digestion stage.

Overall, most of the studied PCs (except for QUE, EGCG, and EA) showed a B% above 50%, after simulated intestinal digestion, with PH (51.55%) showing the lowest value. PG, DAID, and APG (159.82, 158.08, and 147.50%) seemed to be the most bioaccessible compounds after gastrointestinal digestion. Before UHPLC-DAD-MS^n^ analysis, the enzymes in each sample were precipitated through methanol addition (in a proportion of 1:1, v/v). In the following paragraphs, we discuss the bioaccessibility of each compound.

#### 3.1.1 Flavonols

Concerning QUE bioaccessibility (Fig. 4a)), the content after oral phase digestion was 57.23% of the initial concentration, whereas gastric and intestinal percentages were 6.87% and 18.36%, respectively. These results showed a significant effect of SGD, being the B% of the oral phase significantly lower than the initial solution, and higher than gastric and intestinal B%, which are not significantly different. The UHPLC-DAD-MS^n^ analysis of QUE samples along the gastrointestinal digestion has not allowed the detection of any metabolites from enzymatic degradation of QUE. Furthermore, there is no consensus in the literature about the irregular QUE bioaccessibility along the gastrointestinal tract environment. On the one hand, the alkaline pH of the intestine has been seen as the reason for QUE's low bioaccessibility at that stage, resulting in its degradation [34,35]. On the other hand, previous studies have reported that the pH-dependent solubility of QUE may affect its bioaccessibility or intestinal absorption [[36], [37], [38]]. This is because QUE solubility in low pH value of simulated gastric digestion is lower than that of neutral pH value of simulated intestinal digestion. Specifically, QUE solubility is 5.5 and 28.9 μg mL^−1^ at pH 1.5 and 7.5, respectively [36]. This last observation might explain the behavior of QUE at the end of the gastric digestion phase. In fact, previous studies of QUE bioavailability through an *in vitro* gastrointestinal digestion simulation, have not included the oral digestion phase or did not determine QUE oral bioavailability [[39], [40], [41], [42]]. Although according to Gao et al. [36], QUE oral bioavailability is low due to its reduced solubility in water.

The results shown in Fig. 4b demonstrate that RUT B% were above 80% during oral, gastric, and intestinal digestion (86.33, 98.24, and 81.45%, respectively), with no significant differences. These results indicate that *in vitro* SGD did not significantly affect the bioaccessibility of RUT. On the one hand, we anticipated that glycosylation would increase RUT solubility, while on the other hand, we expected enzymatic hydrolysis to occur [32], although the present study did not confirm this. These findings are consistent with those of Gayoso et al. [33], who studied RUT bioaccessibility through an *in vitro* digestion simulator system, demonstrating that RUT remained bioaccessible during gastrointestinal digestion, and the enzymes did not affect its stability.

#### 3.1.2 Flavanones

The B% of NAR and NARN were not different along the simulated gastrointestinal tract (Fig. 4c) and d)). As mentioned, the B% after oral, gastric, and intestinal digestion of aglycone - NAR (97.26, 79.09, and 65.15%, respectively) were lower than those of its glycoside – NARN (133.2, 145.2, and 139.68%, respectively). The B% higher than 100% in this case must probably be related with higher solubility under gastrointestinal conditions compared to the initial solution of glycoside – NARN.

The B% of NAR during oral, gastric, and intestinal phases were lower than the controls without enzymes (102.62, 84.37, and 68.99%, respectively, Table A.3). However, these differences are not statistically significant, which leads to conclude that NAR B% is not affected by an enzyme-NAR interaction. As previously mentioned, the high B% of NARN could be attributed to its glycosylated nature, which may improve the solubility [32]. Furthermore, UHPLC-DAD-MS^n^ analysis did not detect any metabolites of NARN enzymatic digestion. These are the first reported data on pure NAR and NARN bioaccessibility along the gastrointestinal tract using an *in vitro* simulation system.

#### 3.1.3 Flavan-3-ols

The bioaccessibility of EGCG showed variation along the gastrointestinal tract (Fig. 4e)). The EGCG content after the oral phase digestion was low (38.16% of the initial solution) probably due to the neutral pH, which then increased during the gastric phase (71.21%) due to the low pH. The increase of pH value in the intestinal phase led to an EGCG B% decrease, to only 15.09% of the initial solution. In a previous study, Gómez-Mascaraque et al. [43] found that the formation of EGCG dimeric species compromised its bioavailability and proposed encapsulating it in gelatin micro-hydrogels as a strategy to reduce oligomerization. However, the UHPLC-MS/MS results reported that led to this conclusion should be interpreted with caution, as the ion source may itself form dimeric species, resulting in the detection of both [M − H]^-^ and [2M − H]^-^ ions at the same retention time. In fact, the formation of oligomeric species during *in vitro* gastrointestinal digestion simulation is not expected. In order to clarify this, we analyzed the digested samples using UHPLC-DAD-MS^n^ and no metabolites were found, suggesting the hypothesis of an EGCG-enzyme interaction. The results revealed no difference between EGCG controls and enzymatically treated samples regarding bioaccessibility in the oral, gastric, and intestinal phases (34.13, 61.53 and 5.03%, respectively, for controls, Table A.3, and 38.16, 71.21 and 15.09%, respectively, for treated samples). Thus, the variation in EGCG bioaccessibility during *in vitro* simulated digestion is likely due to solubility changes at different pH values in each digestion stage.

#### 3.1.4 Flavones

APG bioaccessibility was different along the gastrointestinal tract (Fig. 4f)). Its B% after gastric digestion (27.60%) was significantly lower than the values obtained after simulated intestinal and oral digestion phases (147.20 and 127.10%, respectively). This behavior may be justified by the phenolic compounds solubility constraining caused by the low pH in gastric phase, while neutral/alkaline pH increases significantly their solubility, which might justify values higher than 100%. As the APG B% in the gastric phase (27.60%) was significantly higher than those of the respective control without enzymes (15.20%, *p* < 0.05, Table A3), the possibility of an enzyme-APG interaction, namely with pepsin, was discarded, confirming the effect of the low pH. Marina et al. [27] have also reported that APG bioaccessible fraction was 2-fold higher after intestinal digestion than the gastric phase due to its higher solubility in alkaline media.

#### 3.1.5 Isoflavones

Worth noting, DAID B% along oral, gastric and intestinal phases (119.06, 81.78, and 158.08%, respectively) (Fig. 4g)) seem to have a similar behavior to that observed for APG. However, DAID B% were not significantly different along the gastrointestinal tract, according to the one-way ANOVA Bonferroni post-hoc test. In addition, according to the *t*-test, there was no difference between DAID samples and control B% along the gastrointestinal tract (78.35, 70.95, and 138.68%, respectively, Table A3). Thus, interactions between DAID and enzymes were not responsible for this variation. So far, and to the best of our knowledge, there is no reported data in the literature about pure DAID bioaccessibility through *in vitro* SGD.

#### 3.1.6 Dihydrochalcones

PH B% after intestinal digestion phase (51.55%), represented in Fig. 4h, was significantly lower comparing with that of simulated oral digestion (96.46%). However, the chromatographic profiles of PH have not changed, and no PH metabolites were detected. Moreover, PH B% in oral, gastric, and intestinal digestion phases and those of respective controls (111.74, 91.73, and 58.91%, respectively, Table A3) were not different. So far, there has been no published data about pure PH bioaccessibility through an *in vitro* simulation of gastrointestinal digestion.

#### 3.1.7 Phlorotannins monomeric units

Similarly, as far as we can ascertain, there is no published data about PG bioaccessibility. Its B% (Fig. 4i)) increased significantly in the intestinal phase of simulated digestion (159.82%) due to the alkaline pH when compared with that of the oral and gastric phases (92.78 and 94.09%, respectively), which were not different from each other. However, comparing its control B% after oral, gastric and intestinal simulated digestion (94.08, 91.32, and 94.91%, respectively, Table A3) with those of enzymatically digested samples, only the intestinal B% were different, according to the *t*-test. Since the intestinal bioaccessibility of the enzymatically digested sample was higher than that of the control, increasing the pH did not have the same effect on PG solubility in the absence of enzymes.

#### 3.1.8 Phenolic acids

The most challenging bioaccessibility screening along the simulated gastrointestinal tract was that of EA (Fig. 4j), which was hindered by its low solubility and extensively seemed to be compromised by the pH of the simulated gastric digestion phase. Some of the samples of EA were below the LOD and LOQ, which inhibited their quantification and the calculation of B% at each phase. Similarly, EA controls (without enzymes) were also not possible to quantify (< LOD and LOQ). In previous studies [25,26], EA bioaccessibility (studied using *Plinia jaboticaba* peel and seed or *Carissa macrocarpa* extracts) along the gastrointestinal tract has been mainly associated with the release and degradation of insoluble ellagitannins from complex matrices at alkaline pH. However, to the best of knowledge, this is the first study of pure EA bioaccessibility through an *in vitro* SGD, so these bioaccessibility differences along the simulated gastrointestinal tract should most likely be due to the low solubility of EA.

### 3.2 Simulation of intestinal absorption using a dialysis membrane

Following digestion from the mouth to the small intestine, the dietary PCs could be either absorbed into the circulatory system or followed to the colon (large intestine), where they might be metabolized by gut microbiota [44]. The structure features of PCs, such as glycosylation, molecular weight, and esterification, influence their absorption in the small intestine [45]. Some studies reported that PCs are only absorbed by 5–10% in the small intestine [28,44].

Herein, we used a dialysis membrane to simulate intestinal absorption of PCs. The samples obtained from internal part of the membrane of QUE, EA, EGCG, APG, and DAID had concentrations below the detection limit, indicating very high intestinal absorption. As represented in Fig. 5, the IA% range of the remaining compounds was between 80 and 95%, so they also seemed to be highly absorbed at the intestine using the dyalisis system. As dialysis relies separation on selective diffusion of molecules across a semi-permeable membrane to separate molecules based on size, this means that for the used membrane that mimicks intestine membrane, there is no significant impediment to the size of tested PCs. PH seemed to be the PC with the lowest IA% (82.28%), whereas RUT, NAR, PG, and NARN presented absorptions higher than 90% (90.79, 92.49, 93.34, and 95.08%, respectively).

As mentioned before, the PCs' structure influences their intestinal absorption. For example, among the different PC classes, isoflavones showed to be most easily absorbed, followed by flavan-3-ols, flavanones, and flavonols glycosides [44]. This last observation is consistent with that observed for DAID, an isoflavone. The amount that passes through the dialysis membrane seemed to be higher than that of NAR, NARN (flavanones), and RUT (flavonol glycoside). However, this system is not considering other events that may exist in the gut, such as enzyme activity or active transport, which may transform the compounds or reduce the bioavailability.

The small intestine enzymatically hydrolyzes flavonoid glycosides, facilitating their absorption [46]. However, the absorption of glycosides varies between different flavonoid classes due to the differences in the hydrolysis process [47]. In this *in vitro* gastrointestinal digestion simulation, verifying the hydrolysis process of RUT and NARN was impossible. Furthermore, gallic acid esterification appeared to hinder the intestinal absorption of flavan-3-ols compared to non-esterified structures [47].

Notwithstanding, the results presented here suggest that the studied PCs are highly absorbable in the simulated small intestine, with greater than 80% absorption rates, when only is considered the effect the size, structure and passive diffusion. Previous studies have suggested that enzyme interactions with PCs may cause their content inside the dialysis membrane to be underestimated [33]. This study found no direct effect of enzyme-PC interactions on the IA% of the studied PCs. Specifically, there were no differences between the absorption percentages obtained for digested samples and their respective controls for RUT (91.63 and 94.75%, respectively), NAR (92.49 and 98.67%, respectively), PH (82.28 and 89.89%, respectively), and PG (93.34 and 95.45%, respectively). These results indicate no direct effect of the interaction between these PCs and enzymes. However, NARN IA% (95.08%) was lower than the respective control (98.60%), suggesting a possible interaction with enzymes.

Moreover, as a simulation of intestinal absorption, dialysis requires careful interpretation since it could be influenced by different factors, mainly compound structure (e.g., molecular weight, sugar units) or dialysis procedure [33]. In particular, the dialysis volumes could also influence the amount of PC that passes the membrane [33]. Therefore, a careful interpretation of these IA% is required. Gayoso et al. [33] have also evaluated the intestinal absorption of RUT after an *in vitro* gastrointestinal digestion using a dialysis membrane. Similarly, to the results obtained in the present study, RUT was highly dialysate through the membrane since the percentage collected from inside of dialysis membrane was 1.65% and thus, indicating an IA% of about 98%. However, the volumes used in this dialysis system were not in the description, which hinders the comparison of how dialysis volumes could influence these results.

In the present study, PCs intestinal absorption evaluation was carried out in the absence of intestinal epithelial cells, namely Caco-2 cell line, which is a model of the intestinal barrier, approved by the FDA Biophamarceutical Classification system, that allows to evaluated the transepithelial transport [48]. Through this Caco-2 monolayer assay, it is possible to determine apparent permeability coefficients (P_app_), which have shown to be highly correlated with *in vivo* human absorption [49], since this cell line enable the transport through diverse mechanisms (namely, passive diffusion, efflux transporters, among others …) [50]. Some of the studied PCs intestinal absorption were already evaluated through the determination of P_app_ for the Caco-2 monolayers, for example, QUE [49,51,52], RUT [51,53], NAR [52,54,55], NARN [56], APG [57], DAID [58] and PH [50].

Most of these studies evaluated the transport of PCs in both directions of Caco-2 cell monolayers – apical-to-basolateral and basolateral-to-apical – to understand how these compounds are bi-directionally transported [51]. Considering the P_app_ values of apical-to-basolateral direction determined in some of these studies, the evaluated PCs, in particular, QUE, RUT and PH, were described as low-permeable compounds [[50], [51], [52]], and when the ratio between P_app_ values from basolateral-to-apical and from apical-to-basolateral is higher than 1.5 may indicate an active efflux transport [[49], [50], [51], [52]]. Notwithstanding, their permeability is highly dependent of diverse factors, namely compounds structure, solubility, stability, concentration, intestinal pH among others [50,52,54,56]. Tourniaiere et al. (2005) determinate a low P_app_ value of apical to basolateral direction for NARN, which seems to be due to its rhamnoglucosyl unit that enterocytic enzymes do not hydrolize, so NARN can be absorbed only after gut microbiota metabolization. In addition, Chabane et al. 2009, showed that NAR is more absorbed in intestine than QUE, since NAR is a more water-soluble than QUE. Moreover, the number of hydroxyl group and the compound configuration are relevant features for its intestinal absorption, thus the five hydroxyl groups and planar configuration of QUE seemed to hinder its permeability on cell membrane, when compared to NAR, which has three hydroxyl groups and more tilted configuration [52]. Therefore, further studies of the intestinal absorption of the studied PCs should be done through the Caco-2 cell monolayers permeability assay.

## 4 Conclusions

The studied PCs did not undergo enzymatic digestion after INFOGEST, as we did not detect any resultant metabolites. The B% determined after the intestinal digestion phase showed that most of the studied PCs were about 50% bioaccessible. However, the solubility of PCs could limit their bioaccessibility, as QUE and EA demonstrated lower bioaccessibility along the simulated gastrointestinal tract due to their low solubility in water. This information is essential for taking advantage of their biological activity to promote human health, specifically in preventing intestinal diseases or at a systemic level. In fact, as a perspective for future work, these results could be quite relevant in the development of nutraceuticals/supplements formulations using these individual components. Additionally, considering that absorption in the gut was simulated using dialysis membrane, all studied PCs showed high absorption rates (>80%), suggesting that they can be absorbed into the systemic circulation or follow to the colon, where gut microbiota may metabolize them.

Furthermore, this work, which consists of a bioaccessibility screening of pure PCs, could complement the literature data. Most published studies concern the evaluation of PCs' bioavailability or bioaccessibility in a complex matrix (food, plant, or extract), which hinders the evaluation of each compound's behavior along the gastrointestinal tract due to some interactions between matrix components. Therefore, these findings can provide essential support for future studies evaluating the potential human health benefits of combining promising PCs or plant-based extracts with a similar composition or enriched ones.

Finally, as an *in vitro* static digestion simulator system, INFOGEST could be seen as a simplistic simulation of gastrointestinal digestion. However, it is a standardized, reproducible system that gives an overview of PCs' bioaccessibility, considering the difference in process complexity between *in vitro* and *in vivo* when interpreting these results. Therefore, it is important to keep in mind that even though these results may not be comparable with other studies where the bioaccessibility and bioavailability of PCs were evaluated using complex food matrices which may account different component interactions, in the present study it was achieved information of some individual PCs' bioaccessibility that were not reported in literature studies until now. Thus, this study could be a building block for further research studies.

## Funding

This work was developed within the scope of the project CICECO-Aveiro Institute of Materials, UIDB/50011/2020, UIDP/50011/2020 & LA/P/0006/2020, financed by national funds through the 10.13039/501100001871FCT/10.13039/501100006111MCTES (PIDDAC). Thank the CBQF for its institutional support through the 10.13039/501100001871FCT project UIDB/50016/2020. Acknowledgments are also due to 10.13039/501100001871FCT/10.13039/501100006111MCTES for the PhD grant to Adriana C.S. Pais (SFRH/BD/143348/2019) and for the research contract under Scientific Employment Stimulus to Sónia A.O. Santos (2021.03348.CEECIND).

## Data availability statement

Data will be made available on request.

## CRediT authorship contribution statement

**Adriana C.S. Pais:** Writing – original draft, Investigation, Formal analysis. **Ezequiel R. Coscueta:** Writing – review & editing, Validation. **Maria Manuela Pintado:** Writing – review & editing, Supervision, Funding acquisition. **Armando J.D. Silvestre:** Writing – review & editing, Supervision, Funding acquisition. **Sónia A.O. Santos:** Writing – review & editing, Supervision, Funding acquisition, Conceptualization.

## Declaration of competing interest

The authors declare the following financial interests/personal relationships which may be considered as potential competing interests:Sonia A.O. Santos reports financial support was provided by Foundation for Science and Technology. Adriana Pais reports financial support was provided by Foundation for Science and Technology. If there are other authors, they declare that they have no known competing financial interests or personal relationships that could have appeared to influence the work reported in this paper.
