# Supplementary material for: Exploring the bioaccessibility and intestinal absorption of major classes of pure phenolic compounds using in vitro simulated gastrointestinal digestion
Source: Heliyon. 2024 Mar 27;10(7):e28894. doi: 10.1016/j.heliyon.2024.e28894 (PMC11016601; doi:10.1016/j.heliyon.2024.e28894)
Supplement: Multimedia component 1 [file mmc1.docx]

# Exploring the Bioaccessibility and Intestinal Absorption of Major Classes of Pure Phenolic Compounds using *In Vitro* Simulated Gastrointestinal Digestion

Adriana C. S. Pais^a^, Ezequiel R. Coscueta^b^, Maria Manuela Pintado^b^, Armando J. D. Silvestre^a^, Sónia A. O. Santos^a,*^

^a^ CICECO-Aveiro Institute of Materials, Chemistry Department, University of Aveiro, 3810-193 Aveiro, Portugal

^b^ Universidade Católica Portuguesa, CBQF - Centro de Biotecnologia e Química Fina – Laboratório Associado, Escola Superior de Biotecnologia, Rua Diogo Botelho 1327, 4169-005 Porto, Portugal

[*santos.sonia@ua.pt](mailto:*santos.sonia@ua.pt)

**Table A.1** - Calibration data used for the UHPLC-UV quantification of phenolic compounds samples from *in vitro* simulated gastrointestinal digestion.

| Phenolic compound | Rt (min) | [M-H]^-^ | λ (nm)^1^ | Concentration range  (μg mL^-1^) | Linear regression equation^2^ | *R*^2^ | LOD ^3^  (μg mL^-1^) | LOQ ^4^  (μg mL^-1^) |
| --- | --- | --- | --- | --- | --- | --- | --- | --- |
| QUE | 17.29 | 301 | 370 | 0.25-20 | $y=1695.6x+4366.7$ | 0.9961 | 1.77 | 5.36 |
| RUT | 12.23 | 609 | 333 | 0.25-40 | $y=1074.6x+914.53$ | 0.9995 | 1.21 | 3.66 |
| NAR | 19.39 | 271 | 285 | 0.25-40 | $y=2582.6x+288.45$ | 0.9992 | 1.37 | 4.14 |
| NARN | 14.72 | 579 | 285 | 0.25-40 | $y=1205.2x+209.56$ | 0.9996 | 1.04 | 3.14 |
| EGCG | 9.45 | 457 | 270 | 0.25-40 | $y=695.1x+1147.7$ | 0.9985 | 1.81 | 5.48 |
| APG | 19.44 | 269 | 333 | 0.5-30 | $y=2895x+3778.4$ | 0.9999 | 0.59 | 1.79 |
| DAID | 16.13 | 253 | 248 | 0.5-30 | $y=4829x+3428.6$ | 0.9996 | 0.85 | 2.56 |
| PH | 19.96 | 273 | 285 | 0.25-60 | $y=2650.4x+223.85$ | 0.9996 | 1.41 | 4.26 |
| PG | 1.37 | 125 | 270 | 0.25-100 | $y=80.756x+213.66$ | 0.9998 | 1.73 | 5.25 |
| EA | 11.65 | 301 | 370 | 0.25-5 | $y=1214.1x+3740.9$ | 0.9585 | 1.56 | 4.72 |

APG – apigenin; DAID – daidzein; D – dialysis phase; EGCG – epigallocatechin gallate; G – gastric phase; IS – initial solution; I – intestinal phase; IA – intestinal absorption; NAR – naringenin; NARN – naringin; O – oral phase; PG – phloroglucinol; PH – phloretin; QUE – quercetin; RUT -rutin; ^1^ Wavelength used in the UHPLC-UV quantification, ^2^ y, and x represent peak area and concentration in μg mL^-1^, respectively, ^3^ LOD - limit of detection, ^4^ LOQ - limit of quantification

**Table A.2** – Concentrations and bioaccessibility percentages of phenolic compounds samples at each phase of the simulated *in vitro* gastrointestinal digestion.

|  | QUE | RUT | NAR | NARN | EGCG | APG | DAID | PH | PG | EA |
| --- | --- | --- | --- | --- | --- | --- | --- | --- | --- | --- |
| Concentration (μg mL^-1^) | | | | | | | | | |  |
| IS | 39.89 | 326.19 | 357.76 | 251.44 | 2068.93 | 9.33 | 18.17 | 288.39 | 5218 | * |
| O | 22.83 ± 2.78 | 281.60 ± 67.69 | 347.95 ± 19.67 | 334.93 ± 40.32 | 789.50 ± 253.03 | 11.86 ± 0.11 | 21.63 ± 5.52 | 278.19 ± 30.82 | 4841.66 ± 308.60 | 4.78 ± 1.75 |
| G | 2.74 ± 0.63 | 320.45 ± 11.33 | 282.96 ± 24.94 | 365.14 ± 71.37 | 1473.22 ± 65.28 | 2.58 ± 0.47 | 14.86 ± 1.98 | 210.15 ± 21.97 | 4910.12 ± 200.81 | * |
| I | 7.33 ± 0.10 | 265.68 ± 13.37 | 233.10 ± 102.39 | 351.21 ± 53.27 | 312.22 ± 46.76 | 13.74 ± 1.22 | 28.72 ± 2.03 | 148.67 ± 32.07 | 8339.90 ± 994.21 | 5.20 ± 0.10 |
| D | * | 22.24 ± 1.52 | 17.50 ± 3.80 | 17.29 ± 1.75 | * | * | * | 26.35 ± 10.09 | 555.31 ± 145.46 | * |
| Bioaccessibility (%) | | | | | | | | | |  |
| IS | 100 ^a^ | 100 ^a^ | 100 | 100 ^a^ | 100 ^a^ | 100 ^b^ | 100 ^a^ | 100 ^a^ | 100 ^b^ | * |
| O | 57.23 ± 6.98 ^b^ | 86.33 ± 20.75 ^a^ | 97.26 ± 5.50 | 133.20 ± 16.03 ^a^ | 38.16 ± 12.23 ^b^ | 127.10 ± 1.17 ^a,b^ | 119.06 ± 30.41 ^a^ | 96.46 ± 10.69 ^a^ | 92.78 ± 5.91 ^b^ | * |
| G | 6.87 ± 1.57 ^c^ | 98.24 ± 3.47 ^a^ | 79.09 ± 6.97 | 145.22 ± 28.38 ^a^ | 71.21 ± 3.16 ^a^ | 27.60 ± 5.02 ^c^ | 81.78 ± 10.87 ^a^ | 72.87 ± 7.62 ^a,b^ | 94.09 ± 3.85 ^b^ | * |
| I | 18.36 ± 0.26 ^c^ | 81.45 ± 4.10 ^a^ | 65.15 ± 28.62 | 139.68 ± 21.19 ^a^ | 15.09 ± 2.26 ^b^ | 147.20 ± 13.05 ^a^ | 158.08 ± 11.20 ^a^ | 51.55± 11.12 ^b^ | 159.82 ± 19.05 ^a^ | * |
| IA | * | 91.63 ± 0.28 | 92.49 ± 5.91 | 95.08 ± 0.66 | * | * | * | 82.28 ± 10.52 | 93.34 ± 2.28 | * |

APG – apigenin; DAID – daidzein; D – dialysis phase; EGCG – epigallocatechin gallate; G – gastric phase; IS – initial solution; I – intestinal phase; IA – intestinal absorption; NAR – naringenin; NARN – naringin; O – oral phase; PG – phloroglucinol; PH – phloretin; QUE – quercetin; RUT -rutin; * < LOD and/or LOQ; Standard deviation values were calculated from the three replicates analyzed in duplicate. Different letters indicate significant differences (*p* < 0.05), determined by one-way ANOVA followed by Bonferroni’s post-hoc test.

**Table A.3** – Concentrations and bioaccessibility percentages of phenolic compounds controls (without enzymes) at each phase of the simulated *in vitro* gastrointestinal digestion.

|  | QUE | RUT | NAR | NARN | EGCG | APG | DAID | PH | PG | EA |  |
| --- | --- | --- | --- | --- | --- | --- | --- | --- | --- | --- | --- |
| Concentration (μg mL^-1^) | | | | | | | | | | | |
| IS | 39.89 | 326.19 | 357.76 | 251.44 | 2068.93 | 9.33 | 18.17 | 288.39 | 5218 | * |  |
| O | 13.64** | 331.30 ± 8.52 | 367.13 ± 4.00 | 322.30 ± 36.59 | 706.03 ± 39.46 | 12.60 ± 0.76 | 14.24 ± 4.52 | 322.24 ± 20.85 | 4909.58 ± 96.26 | * |  |
| G | * | 317.06 ± 7.46 | 301.86 ± 16.45 | 332.53 ± 16.91 | 1272.99 ± 111.59 | 1.42 ± 0.28 | 12.89 ± 0.67 | 264.55 ± 38.18 | 4765.51 ± 197.78 | * |  |
| I | 7.02 ± 0.7 | 285.21 ± 19.38 | 246.82 ± 120.35 | 319.15 ± 27.85 | 104.15 ± 16.86 | 14.78 ± 0.47 | 25.20 ± 1.64 | 169.91 ± 46.69 | 4952.79 ± 277.44 | * |  |
| D | * | 15.12 ± 5.54 | 3.29 ± 0.96 | 4.46 ± 0.44 | * | 2.88 ± 0.19 | * | 17.18 ± 2.01 | 225.29 ± 125.33 | * |  |
| Bioaccessibility (%) | | | | | | | | | | | |
| IS | 100 | 100 ^a,b^ | 100 | 100 ^a^ | 100 ^a^ | 100 ^c^ | 100 ^a^ | 100 ^a,b^ | 100 ^a^ | * |  |
| O | 34.19* | 101.57 ± 2.61 ^a^ | 102.62 ± 1.12 | 128.18 ± 14.55 ^a^ | 34.13 ± 1.91 ^c^ | 134.98 ± 8.17 ^b^ | 78.35 ± 24.88 ^a^ | 111.74 ± 7.23 ^a^ | 94.08 ± 1.84 ^a^ | * |  |
| G | * | 97.20 ± 2.29 ^a,b^ | 84.37 ± 4.60 | 132.25 ± 6.73 ^a^ | 61.53 ± 5.39 ^b^ | 15.20 ± 3.04 ^d^ | 70.95 ± 3.71 ^a^ | 91.73 ± 13.24 ^a,b^ | 91.32 ± 3.79 ^a^ | * |  |
| I | 17.59 ± 1.70 | 87.44 ± 5.94 ^b^ | 68.99 ± 33.64 | 129.93 ± 11.08 ^a^ | 5.03 ± 0.81 ^d^ | 158.32 ± 5.05 ^a^ | 138.68 ± 9.01 ^a^ | 58.91 ± 16.19 ^b^ | 94.91 ± 5.32 ^a^ | * |  |
| IA | * | 94.75 ± 1.72 | 98.67 ± 1.47 | 98.60 ± 0.14 | * | 80.49 ± 1.28 | * | 89.89 ± 3.20 | 95.45 ± 2.64 | * |  |

APG – apigenin; DAID – daidzein; D – dialysis phase; EGCG – epigallocatechin gallate; G – gastric phase; IS – initial solution; I – intestinal phase; IA – intestinal absorption; NAR – naringenin; NARN – naringin; O – oral phase; PG – phloroglucinol; PH – phloretin; QUE – quercetin; RUT -rutin; * < LOD and/or LOQ; Standard deviation values were calculated from the three replicates analyzed in duplicate. Different letters indicate significant differences (*p* < 0.05), determined by one-way ANOVA followed by Bonferroni’s post-hoc test.
